# Molecular Inversion Probe: A New Tool for Highly Specific Detection of Plant Pathogens

**DOI:** 10.1371/journal.pone.0111182

**Published:** 2014-10-24

**Authors:** Han Yih Lau, Ramkumar Palanisamy, Matt Trau, Jose R. Botella

**Affiliations:** 1 Plant Genetic Engineering Laboratory, School of Agriculture and Food Sciences, University of Queensland, Brisbane, Queensland, Australia; 2 Australian Institute for Bioengineering and Nanotechnology, University of Queensland, Brisbane, Queensland, Australia; Virginia Tech, United States of America

## Abstract

Highly specific detection methods, capable of reliably identifying plant pathogens are crucial in plant disease management strategies to reduce losses in agriculture by preventing the spread of diseases. We describe a novel molecular inversion probe (MIP) assay that can be potentially developed into a robust multiplex platform to detect and identify plant pathogens. A MIP has been designed for the plant pathogenic fungus *Fusarium oxysporum* f.sp. *conglutinans* and the proof of concept for the efficiency of this technology is provided. We demonstrate that this methodology can detect as little as 2.5 ng of pathogen DNA and is highly specific, being able to accurately differentiate *Fusarium oxysporum* f.sp. *conglutinans* from other fungal pathogens such as *Botrytis cinerea* and even pathogens of the same species such as *Fusarium oxysporum* f.sp. *lycopersici*. The MIP assay was able to detect the presence of the pathogen in infected *Arabidopsis thaliana* plants as soon as the tissues contained minimal amounts of pathogen. MIP methods are intrinsically highly multiplexable and future development of specific MIPs could lead to the establishment of a diagnostic method that could potentially screen infected plants for hundreds of pathogens in a single assay.

## Introduction

Agriculture is a major economic activity with a total annual value of $1500 billion US dollars. However, up to a third of the agricultural production is lost due to three major causes: disease outbreaks, insect attack and weed competition [1]. Among them, losses caused by crop diseases are the most important issue globally, especially in agriculturally reliant countries. In the absence of resistance, the ideal method to control disease outbreaks is by early detection in the field before it spreads to neighboring farms. It is therefore essential to develop new disease diagnostic technologies that are sensitive, reproducible, highly specific and able to detect multiple pathogens in a single assay.

Numerous methods have been evaluated to diagnose plant diseases and detect plant pathogens [2]. The conventional approaches involve identifying the morphological changes in the plant, followed by culturing the pathogens *in vitro* for further characterization [3]. Despite the high accuracy of these approaches, they are time consuming, labor intensive and most importantly, it requires experienced plant pathologists, a luxury in many developing countries, to identify and classify the plant pathogens responsible for the disease. Hence antibody-based rapid diagnostic approaches such as enzyme-linked immunosorbent assays (ELISAs) [4], [5], immunoblot [6] and immunofluorescent tests [7] have been developed and widely used to identify a number of plant pathogens. However, these antibody-based methods have been reported to be cross-reactive and sometimes yield false-negative results [8]. To increase sensitivity and specificity, DNA based molecular techniques have recently become the most powerful tool in plant disease diagnostics [9], [10]. Amplifying DNA regions unique to a specific pathogen using the polymerase chain reaction (PCR) is one of the most widely used molecular biology techniques and has become a foundation of DNA-based plant pathogen detection. Although PCR based assays demonstrate improved sensitivity and specificity compared to older technologies [11]–[14], they have limited multiplexing capability when detecting and identifying unknown pathogens in diseased plant samples. Indeed, multiplex PCR assays containing multiple primer sets are more prone to non-specific amplification resulting in false positive results [15], [16].

Furthermore, most of the DNA/PCR-based diagnostic methods have been designed to target the internal transcribed spacer (ITS) regions in the ribosomal RNA genes because of their high copy number and the easy access to large amounts of sequence information in databases [17]–[19]. As a result, the ITS region has been widely used to identify and classify plant pathogens [20]. However, the ITS region is highly conserved in same species and therefore not an ideal target region for intra-species pathogen identification such as differentiating between the *conglutinans* and *lycopersici* formae speciales from *Fusarium oxysporum*. Therefore, an assay that can readily screen for a particular pathogen or identify the presence of several pathogens in a given sample with high specificity, sensitivity, reproducibility and also amenable to high-throughput multiplex detection is highly desirable.

Currently the most effective way to identify unknown pathogens still involves symptom observation, characterization of the pathogen and proof of Koch’s postulates which requires one to few weeks to confirm the identity of the pathogen. Herein, we describe a novel method that can screen for pathogens with high specificity and sensitivity using molecular inversion probes (MIPs). Molecular inversion probes have been previously used for various clinical applications such as high-throughput analysis of single nucleotide polymorphisms, DNA methylation, detecting genomic copy number changes and other genotyping applications [21]–[23]. MIPs offer a number of advantages over other genome based PCR technologies. Firstly, the MIP backbone physically restricts the two binding domains for localized interaction which increases the specificity of the detection assay. Secondly, the exonuclease digestion step prior to PCR amplification digests all the non-circularized MIPs further increasing the specificity of the assay. Finally, MIPs are highly multiplexable with several thousands of targets can be interrogated in a single multiplex assay.

In this study, we have used the MIP technology as a diagnostic tool to screen for plant pathogens in infected tissues. Three economically important plant pathogens, including two from the same species, have been selected for this study (*Fusarium oxysporum* f.sp. *conglutinans*, *Fusarium oxysporum* f.sp. *lycopersici* and *Botrytis cinerea*). As proof of concept, we designed a MIP to target a unique sequence present in the *F. oxysporum* f.sp. *conglutinans* (Foc) genome. The specificity, sensitivity and detection limit of the assay were assessed and used to detect the presence of pathogen in infected *Arabidopsis thaliana* samples.

## Materials and Methods

### Plant and pathogen materials

*Arabidopsis thaliana* ecotype Columbia (Col-0) was obtained from the Arabidopsis Biological Resource Center (ABRC; Ohio State University). *Fusarium oxysporum* f. sp. *conglutinans* (Foc), *Fusarium oxysporum* f.sp. *lycopersici* (Fol) and *Botrytis cinerea* (Bc) were obtained from the Department of Agriculture, Fisheries and Forestry, Queensland, Australia.

### *Arabidopsis thaliana* growth

Arabidopsis seeds were sown on soil in a small pot (50 mm diameter) and kept at 4°C for three days before transferring to short day growing conditions (photoperiod 8/16 light/dark; 23°C) for another 7 days. The seedlings were then carefully removed from the soil by immersing in water and transplanting them into trays. The seedlings were grown in short day conditions for an additional 7–14 days until the size of plant reached 25 mm in diameter.

### *Fusarium oxysporum* f.sp. *conglutinans* and *Fusarium oxysporum* f.sp. *lycopersici* cultures preparation

A small agar block containing Foc/Fol hyphae was placed on a plain agar plate and incubated at room temperature for 4–6 days until the hyphae were visible. Three agar blocks (5 mm × 5 mm) were cut from the fresh culture plate and transferred into 200 ml of potato dextrose broth (PDB) in a 1 liter bottle. The culture was incubated at 28°C with shaking at 110 rpm for 3–4 days. The culture was then filtered through 4 layers of Miracloth to separate the mycelia and the spores. The elute containing the spores was used for inoculation while the mycelia were used for genomic DNA extraction. The concentration of spores in elution was quantified using a hemocytometer and diluted with distilled water to a final concentration of 10^6^ spores per ml for inoculation.

### *F.oxysporum* f.sp. *conglutinans* inoculation

A tray containing 20 Arabidopsis seedlings was immersed in water to remove the soil. Then the seedlings were carefully cleaned and dried on a tissue paper before immersing them into inoculation solution for 30 seconds. After the inoculation, the seedlings were transplanted onto soil and grown at 28°C in short day conditions for disease evaluation.

### *Botrytis cinerea* culture preparation

Hyphae of Bc on a small agar block was placed on potato dextrose agar (PDA) and incubated at room temperature for 10–14 days until the agar surface was fully covered by mycelium. The layer containing Bc mycelium on PDA surface was taken and used for genomic DNA extraction.

### Genomic DNA preparation

Foc, Fol and Bc genomic DNA was extracted using the protocol as previously described [24]. Four-days-old Foc, Fol cultures were prepared as described above and filtered with four layers of Miracloth, the mycelium trapped on the Miracloth surface was transferred into mortar and immediately frozen with liquid nitrogen. However, the Bc mycelium was taken directly from the 10–14 days old culture on PDA plate. Approximately 100 mg of the finely ground mycelium tissue was transferred to a 1.5 ml microcentrifuge tube. The ground tissue was mixed with 400 µl of extraction buffer (150 mM Tris base, 2% (w/v) SDS, 50 mM EDTA, 1% (v/v) b-mercaptoethanol, adjusted to pH 7.5 with boric acid) and vortexed for 5 min. The mixture was then added with 100 µl of absolute ethanol and 44 µl of 5 M potassium acetate, the mixture was vortexed for 1 min following the addition of each reagent. The solution was added with 500 µl of chilled chloroform : isoamyl alcohol (24∶1, v/v) and vortexed for 1 min before centrifugation (maximum speed, 3 min at room temperature). Approximately 500 µl of upper phase was transferred to a new 1.5 ml tube and 500 µl of phenol : chloroform : isoamyl alcohol (25∶24:1, v/v) was added. The mixture was again centrifuged at maximum speed for 3 min at room temperature to separate into an upper and a lower phase. The upper phase (400 µl) was transferred to a new tube and mixed well with 1 ml of cold absolute ethanol. The mixture was then incubated at −80°C for 30 min before centrifuging at 4°C for 30 min at 15,000 × g to pellet DNA. The supernatant was removed and the pellet was washed with 80% ethanol. The pellet was resuspended in 100 µl H_2_O and 5 µl of RNase A (10 mg/ml) was added, followed by 37°C incubation for 20 min. After the incubation, 10 µl of 3M sodium acetate and 100 µl of isopropanol were added to the mixture. The genomic DNA was allowed for precipitation at −20°C for 10 min then centrifuged for 2 min at maximum speed. After washing with 70% ethanol, the dried pellet was resuspended in H_2_O. Finally the concentration of extracted genomic DNA was quantified using NanoDrop ND-1000 spectrophotometer (Thermo Scientific, Wilmington, USA).

Genomic DNA from Arabidopsis leaves was extracted using a modified Doyle and Doyle CTAB method [25]. Leaf tissue was collected, frozen with liquid nitrogen and ground into fine powder. Approximately 100 mg of ground leaf tissue was then mixed with 500 µl of pre-heated 50°C extraction buffer (2% w/v CTAB, 1.42 M NaCl, 20 mM EDTA, 100 mM Tris HCl pH 8.0, 1% w/v PVP 40), containing proteinase K (40 µg/ml) and 0.4% β-mercaptoethanol. The mixture was vortexed for 5 min before incubating at 50°C in a water bath for 30 min, inverting every 5 min. The homogenate was then transferred to a 65°C water bath and incubated for another 15 min. The solution was then allowed to cool to room temperature and 500 µl of chilled chloroform : isoamyl alcohol was added (24∶1, v/v). After a vigorous shake, tubes were gently rocked for 15 min at room temperature, followed by centrifugation at 15,000 × g for 10 min. Approximately 400 µl of supernatant was transferred to a new 1.5 ml tube, 800 µl of cold absolute ethanol added and gently inverted to precipitate the genomic DNA. After incubation for 1 h in −80°C, genomic DNA was recovered by centrifugation (30 min, 15,000 × g, 4°C). After washing with 80% ethanol, the precipitate was dried and resuspended in 100 µl of H_2_O. The genomic DNA was treated with RNase A as described above and the concentration was quantified with NanoDrop ND-1000 spectrophotometer.

### Molecular inversion probe assay

#### Design of MIP

**Figure 1:**
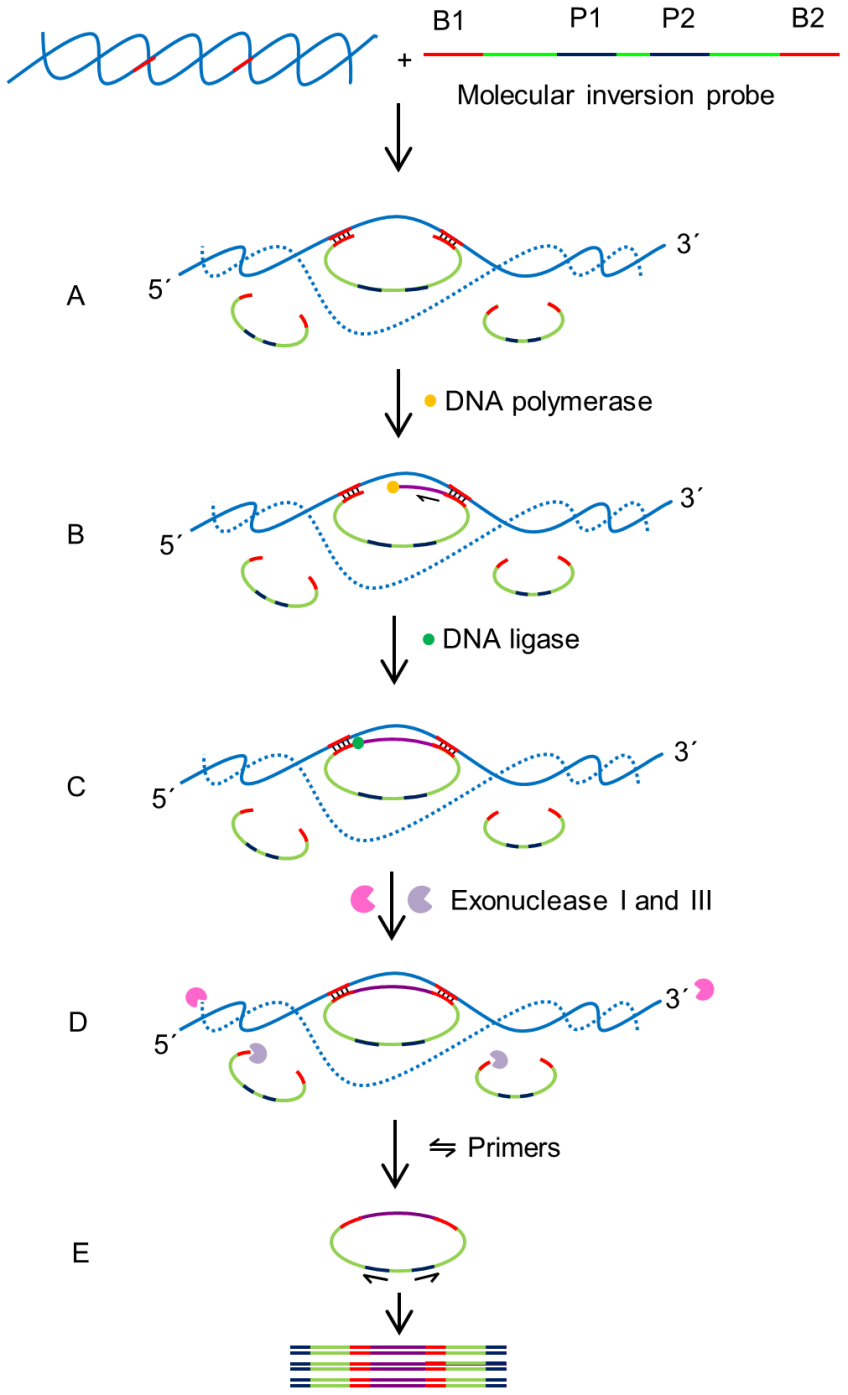
Schematic outline of molecular inversion probe (MIP) assay. The MIP consists of two binding domains at its 3′ and 5′ ends which are designed complementary to target sequences in DNA. The MIPs also contained two universal primer binding domains (P1 and P2) in its DNA backbone. (A) Hybridization: B1 and B2 bind to specific sequences on the target DNA creating a 48 base single stranded gap between the binding domains of the MIP. (B) Gap filling: A DNA polymerase that lacks exonuclease and strand displacement activities synthesizes DNA from 3′ end of the MIP to 5′ end until the single stranded gap is filled. (C) Ligation: A DNA ligase ligates the 3′ end of the newly synthesized DNA strand with the 5′ end of the MIP creating a circular DNA. (D) Digestion: exonucleases I and III digest the linear MIPs and the DNA target in the reaction mixture leaving the circularized MIPs for amplification. (E) Polymerase chain reaction (PCR): A pair of universal primers (P1 and P2) amplifies the circularised MIP using the universal primer binding domains to generate PCR amplicons.

The Foc race 2 genome sequence was obtained from GenBank, National Center for Biotechnology Information (NCBI). Bioinformatic analysis was performed and candidate target Foc sequences were identified. Candidate sequences were subjected to BLAST searches to identify homologous DNA fragments in other organisms. Special attention was devoted to find sequences present in the Foc genome but absent from Fol. A fragment showing no homology to any available sequence in the databank was chosen. Based on the unique sequence, a MIP (named MIPFOC01) was designed with 20 nt and 18 nt of binding regions (B1 and B2, respectively) at 3′ and 5′ end of MIP, respectively. The binding regions sequences were complimentary to the DNA target. The Tm values of both binding regions were adjusted to obtain slightly lower Tm at B1 in order to allow B1 hybridized to DNA target before B2. When the MIP hybridized to the DNA target, it generated a single stranded gap (48 nt) between the two binding domains. The binding regions of MIP were connected with a 104 nt linker sequence containing primer sites (P1 and P2) for sequence amplification (Fig. 1). The MIP had a total size of 143 nt and was analyzed using the OligoAnalyzer 3.0 software (Integrated DNA Technologies) (http://www.idtdna.com/analyzer/Applications/OligoAnalyzer/) to avoid any secondary structures that might interfere with the hybridization process. DNA oligonucleotides listed in Table 1 were purchased from Integrated DNA technologies (IDT, Iowa, USA).

**Table 1:** Oligonucleotide sequences.

MIP for *F.oxysporum* f.sp. *conglutinans*	MIPFOC01	5′-**AGTAGAATGAAGCCTCCCCC**AGGGTTTGTTGTGGTCAGAATTCTGTCTG**ATGGCTCTTCAGTCCTATAACG**UUU**CCAAATGCTGTGTAGGTCATCT**CACCAATGCATACCAGGCTCACTTTGGGA**GGACGAGGGAAAGAGTTG**-3′
Universal primers for amplifying circular MIPs	MIP forward primer(P1)	5′-CGTTATAGGACTGAAGAGCCAT-3′
	MIP reverse primer (P2)	5′-CCAAATGCTGTGTAGGTCATCT-3′
Real-time PCR primers for Foc quantification	Foc-F1	5′-GGGGGAGGCTTCATTCTACT-3′
	Foc-R1	5′-TGGGACGAGGGAAAGAGTTG-3′
	Foc-F2	5′-CAGACTTTCCACAGCAATGCGT-3′
	Foc-R2	5′-CCATGGGACGAATAGGCACC-3′

Nucleotides in bold underlined and double-underlined indicate the target binding sites and the PCR amplification primer sites, respectively.

#### Hybridization

1 pg of MIP was mixed with genomic DNA in 10 µl reactions containing 1X ligase buffer (200 mM Tris-HCl (pH 8.3), 250 mM KCl, 100 mM MgCl_2_, 5 mM NAD, and 0.1% Triton X-100). The genomic DNA was initially denatured at 95°C for 6 min followed by 85°C for 10 min. The temperature was then gradually decreased from 70°C to 56°C at the rate of 1°C/30 sec, followed by 4 h incubation at 56°C.

#### Gap filling and ligation

The gap filling reaction was performed by adding 5 µl of the reaction mixture containing 1 unit ampligase enzyme (Ampligase Thermostable DNA Ligase, Epicentre Biotechnologies, Wisconsin, USA), 1 unit stoffel fragment DNA polymerase (Applied biosystems, USA), 125 nM of each dNTP (Roche diagnostics, Manheim, Germany) in 1X ligase buffer (200 mM Tris-HCl (pH 8.3), 250 mM KCl, 100 mM MgCl_2_, 5 mM NAD, and 0.1% Triton X-100). The reaction mixture was incubated at 56°C for 2 h followed by cycling the reaction for four cycles using the following conditions, initial denaturation at 95°C for 6 min followed by 85°C for 10 min. The temperature was then gradually decreased from 65°C to 56°C at the rate of 1°C/30 sec followed by 4 h incubation at 56°C.

#### Exonuclease digestion

An enzyme mixture containing 10 units of exonuclease I (NEB, Ipswich, USA) and 50 units of exonucelase III (NEB, Ipswich, USA) was added to the gap-fill reaction mixture to digest non ligated MIPs and liner DNA targets. The reaction mixture was initially incubated at 37°C for 60 min followed by heat killing the enzymes at 80°C for 20 min.

#### Polymerase chain reaction (PCR)

Amplification of ligated MIPs was performed in a 60 µl reaction containing 1.5 unit Taq DNA polymerase (AmpliTaq DNA Polymerase, Applied Biosystems, Australia), 0.7X PCR buffer (AmpliTaq 10X PCR buffer), 0.2 mM each dNTP, 0.1% tween 20 and 125 nm of each universal forward and reverse primers (Table 1). PCR reaction was carried out in a Bio-Rad thermo cycler (MJ Mini Personal Thermal Cycler) using the following conditions: denaturation at 94°C for 10 min followed by 50 cycles of 94°C for 30 sec, 65°C for 45 sec and 72°C for 30 sec. The products were analyzed by gel electrophoresis using 2.5% agarose gels in sodium borate buffer. The gel was stained with ethidium bromide (0.5 µg/ml) then visualized under a 254 nm transilluminator (Vision-capt version 14.2) and the DNA band intensities on gel were quantified using ImageJ software. The PCR amplicon was purified using a QIAquick PCR purification kit (QIAGEN Pty. Ltd., Venlo, Netherlands) and quantified using a ND-1000 Nanodrop spectrophotometer (Thermo Scientific, Wilmington, USA). The purified DNA was then used in Sanger sequencing and the sequencing result was analyzed using CLUSTALW multiple sequence alignment (http://www.genome.jp/tools/clustalw/).

### Real-time PCR

Two sets of primers were designed with each targeting a 100 bp region in the Foc genome (Table 1). Real-time PCR was performed using the Rotor-Gene Q-Pure Detection System (Software Ver. 2, Qiagen Inc., Valencia, CA, USA). The standard curve was plotted according to the target DNA concentration against the Threshold cycle (Ct) value observed in the real-time PCR assay. The total reaction volume of 20 µl contained either pure Foc genomic DNA (0.1−20 ng) or 100 ng of genomic DNA from infected plant samples, 250 nM of each primer, 1 unit Taq DNA polymerase (AmpliTaq DNA Polymerase, Applied Biosystems, Australia), 1X PCR buffer (AmpliTaq 10X PCR buffer), 0.2 mM each dNTP, 2 mM magnesium chloride and 5.0 µM SYTO-9 (*Invitrogen*, USA). Real-time PCR reactions were carried out at the following conditions: 95°C for 5 min (denaturation and hot start activation), 35 cycles of 95°C for 30 sec, 58°C for 30 sec, 72°C for 30 sec and a final elongation step at 72°C for 5 min. After the real-time PCR, the temperature increased from 72°C to 95°C to analyze the melting curves of the PCR products.

## Results

### Assay design

A MIP assay was designed to screen and specifically identify Foc infecting *A. thaliana* plants (Fig. 1). The MIP was hybridized to Foc genomic DNA forming a circular loop with a single stranded gap between the two binding regions (Fig. 1A). A *Taq* DNA polymerase, lacking 5′-3′ exonuclease and strand displacement activities, was then used to initiate DNA synthesis from the 3′ end of the MIP in a gap-fill reaction [21]. Following strand synthesis, a DNA ligase was used to ligate the newly synthesized strand with the 5′ end of the binding domain generating a circular DNA molecule. To ensure increased sensitivity, the unligated linear probes were eliminated by digestion with exonuclease. The circularized MIP, which is immune to exonuclease digestion, was amplified in a PCR reaction to detectable levels using universal primers designed for two domains in the MIP backbone. In the absence of the target pathogen DNA, the linear MIP was digested in the exonuclease step and no DNA amplicon was produced.

### Design of the Molecular Inversion Probe

The MIP (MIPFOC01) used in this study was designed to recognize a fragment of Foc genomic DNA between genes encoding for hypothetical proteins EGU89127.1 and EGU89126.1 (GenBank locus AGNF01000001). MIPFOC01 is 143 nucleotides (nt) long oligonucleotide with two binding domains, 20 nt and 18 nt each at the 5′ and 3′ end, respectively (Table 1). The binding domains were designed to be complementary to the target region of interest (Fig. 1). The melting temperature of the 5′ binding domain was designed to be few degree centigrade higher than the melting temperature of the 3′ binding domain to enable the MIP to hybridize to the target DNA and form a loop with a 48 nt single stranded gap in between the two binding regions (Fig. 1A). The backbone of MIPFOC01 was designed manually containing two ‘universal primer’ binding domains that were designed to be present in the backbone of other MIP probes. In this way a single set of primers can be used for amplifying several MIPs, independently of the probe and the pathogen being analyzed.

### Detection limit

During disease evolution, pathogens gradually multiply inside their hosts. On many occasions, by the time the disease symptoms are visible, the pathogen may have already spread to neighboring plants making it crucial to determine the lower detection limit of any diagnostic assay that can reliably generate a detectable signal.

**Figure 2:**
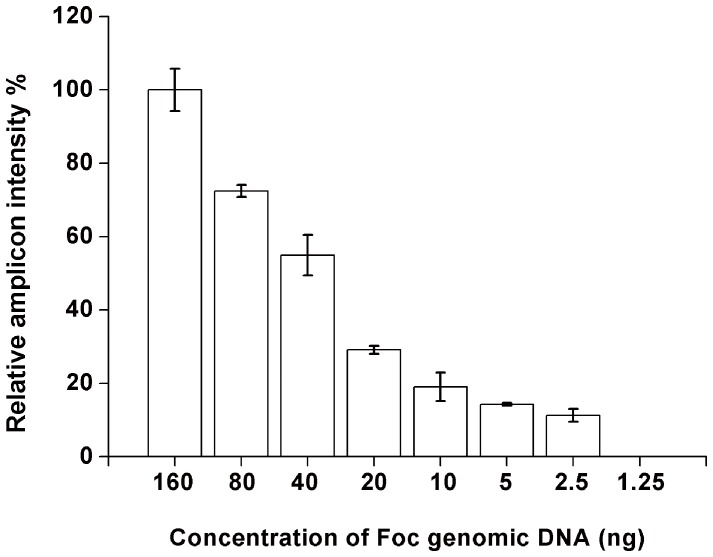
Detection limit of the MIP assay. The MIP (MIPFOC01) was mixed with different amounts of Foc genomic DNA (160 ng, 80 ng, 40 ng, 20 ng, 10 ng, 5 ng, 2.5 ng and 1.25 ng) in triplicates and analyzed in a MIP assay. The PCR amplicon was electrophoresed through an agarose gel and the intensity of the DNA bands were measured. Error bars represent the standard deviation of three replicates.

Genomic DNA was extracted from *in vitro* grown Foc pure cultures and replicate assays containing different amounts of purified Foc genomic DNA ranging from 160 ng to 1.25 ng were performed using MIPFOC01. Following hybridization and gap-fill reaction, the circularized MIPs were amplified by PCR. The PCR amplicons were analyzed by gel electrophoresis and the relative intensities of the DNA bands were measured. As expected, the intensity of the DNA band increased with increasing amounts of genomic DNA in the reaction but signal saturation was not reached even at relatively high concentrations of pathogen genomic DNA (Fig. 2). No bands were observed when the amount of input genomic DNA was decreased below 2.5 ng which was same as the control reaction containing no genomic DNA.

### Sensitivity analysis

Even though detection limits for diagnostic methods are usually determined using pure pathogen DNA [26], [27], in a real world situation DNA extracted from an infected plant contains a complex mixture of genomic DNA from the plant as well as the pathogen. The ratio of plant DNA to pathogen DNA in a given sample is variable as it depends on the pathogen and disease progression, but even in advanced stages of infection, plant DNA will always be present in vast excess over pathogen DNA. Therefore, detecting low number of pathogens present in plant tissues during early disease stages has always been a challenge in disease management.

**Figure 3:**
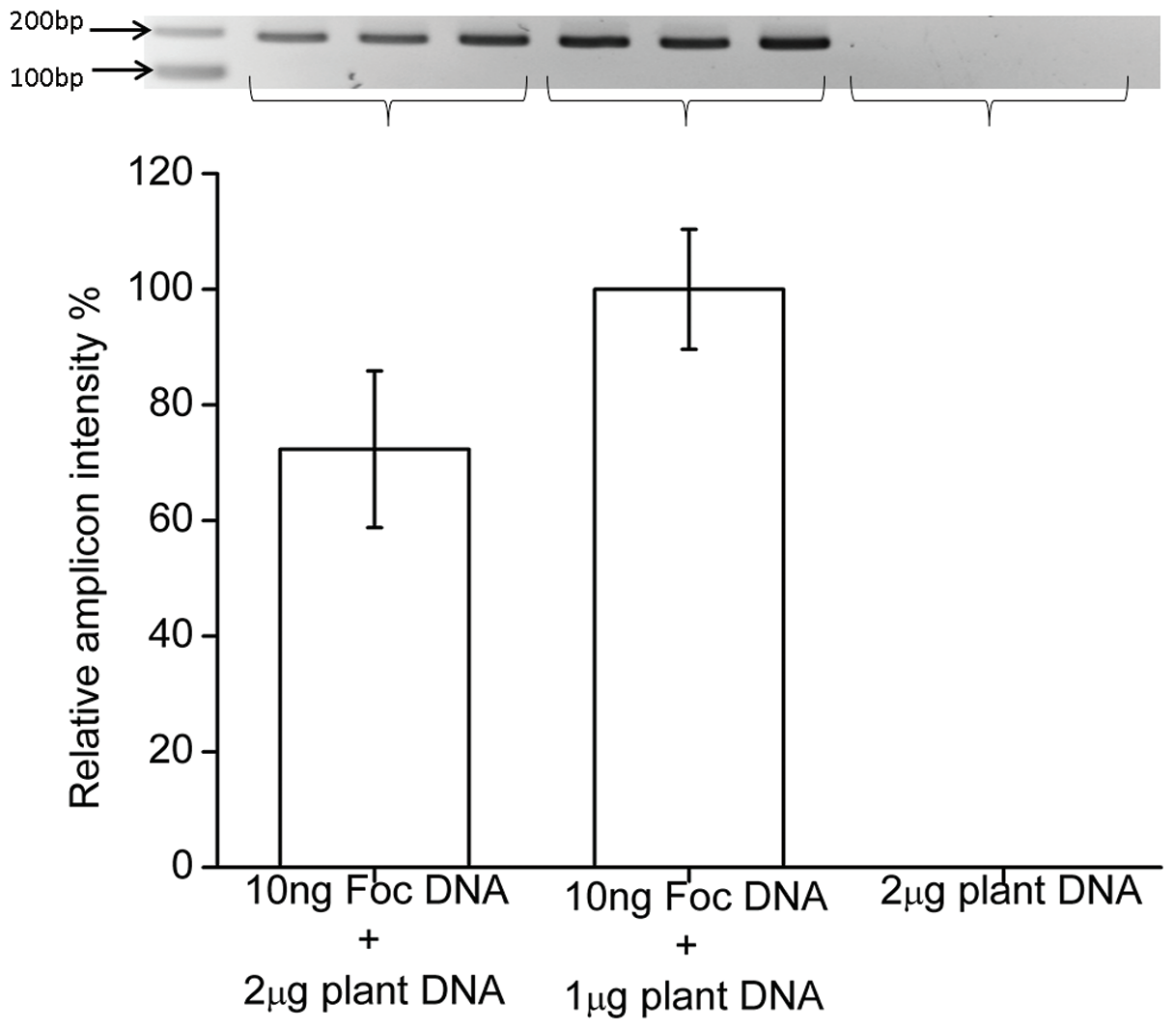
Effect of host DNA on the sensitivity of the MIP assay. Reactions containing 10 ng of Foc genomic DNA were mixed with either 1 µg or 2 µg of genomic DNA from *A. thaliana* in triplicates and analyzed in a MIP assay. A sample containing 2 µg of *A. thaliana* genomic DNA was used as negative control. Error bars represent the standard deviation of three replicates.

To investigate the sensitivity of the MIP assay, increasing amounts of *A. thaliana* genomic DNA were added to triplicate reactions containing MIPFOC01 and 10 ng of Foc genomic DNA. The PCR amplicons were analyzed using gel electrophoresis revealing that band intensity decreased significantly (p = 0.049) as the amount of *A. thaliana* genomic DNA in the reaction increased (Fig. 3). As expected, the control reactions containing Arabidopsis genomic DNA but no Foc genomic DNA failed to generate any PCR product. Our results indicate that the MIP assay using MIPFOC01 is able to detect Foc DNA even in the presence of 200 fold excess of plant DNA.

### Specificity analysis

Specificity, being able to reliably identify a particular pathogen from other species, closely related species or even different races in the same species, is essential for a diagnostic assay. Furthermore, in many instances crops can be infected by multiple pathogens and it is important to avoid false positive results caused by close relatives [28].

**Figure 4:**
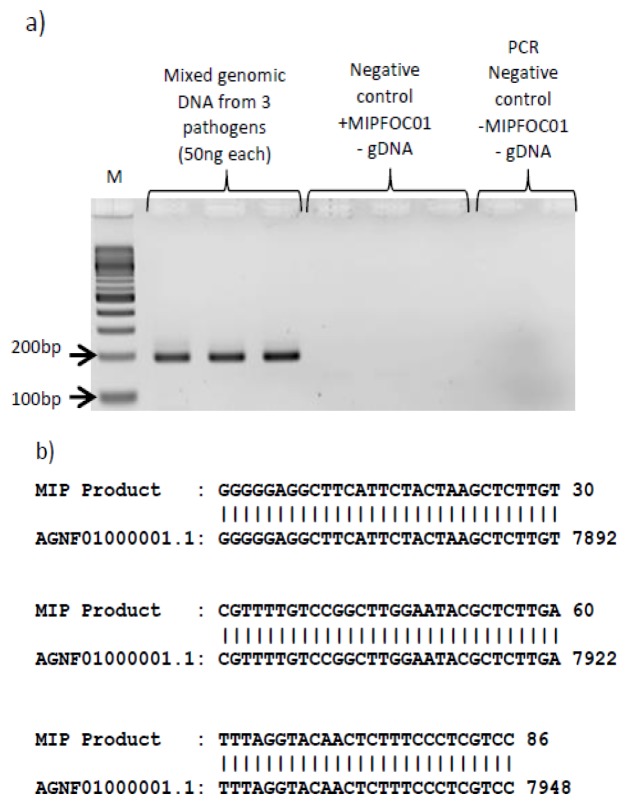
Specificity test of the MIP assay. a) 50 ng of genomic DNA from each of the three infectious plant pathogens (Foc, Fol and Bc) were mixed and analyzed in a MIP assay using MIPFOC01. The PCR products were analyzed using gel electrophoresis. b) The DNA amplicon was purified and sequenced using Sanger sequencing method. The data obtained indicated a perfect match with AGNF01000001.1 (*Fusarium oxysporum* f. sp. *conglutinans* race 2 54008 cont1.1).

The specificity of the MIP assay and the MIPFOC01 probe was assessed by detecting the presence of Foc DNA in a pool containing DNA from two other pathogens; a close relative (*Fusarium oxysporum* f.sp. *lycopersici*) and a non-related necrotrophic fungal pathogen (*Botrytis cinerea*). The MIP assay was performed in triplicates using the DNA mix extracted from the three pathogens as sample and the reaction products analyzed using gel electrophoresis. A single DNA fragment was observed of the expected size (∼200 bp) (Fig. 4a). The DNA amplicon was purified and sequenced revealing an exact match with the targeted Foc genomic sequence over the entire length of the amplicon (191 bp) (Fig. 4b). No products were observed when the MIP assay was performed using MIPFOC01 in the absence of the pathogen DNA mix or when both MIPFOC01 and the pathogen DNA mix were omitted. Additionally, the MIP assay failed to amplify any products when *Fusarium oxysporum* f.sp. *lycopersici* or *Botrytis cinerea* were used as source of DNA (Fig. S1). This result indicates that MIPFOC01 is highly specific for Foc even in the presence of other pathogens from the same species.

### Disease development and sampling

*Arabidopsis thaliana* seedlings were manually inoculated with Foc as described by Trusov *et al.* [29]. Inoculated plants started to show chlorosis in leaves and wilt symptoms 7 days after inoculation (dai), and the disease progressed until the fungus invaded all tissues killing the plant 21−25 dai. Non-inoculated control plants stayed healthy and no abnormal symptoms were observed over the experimental period.

**Figure 5:**
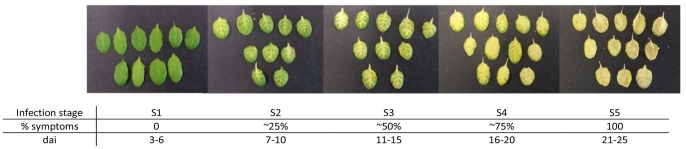
Disease symptom development and classification. The *A. thaliana* leaves were checked for visible symptoms from 1 day after inoculation (dai) until 25 dai and classified into 5 infection stages according to their severity. Stage 1∶0% symptom severity (3–6 dai); Stage 2: ∼25% symptom severity (7–10 dai); Stage 3: ∼50% symptom severity (11–15 dai); Stage 4: ∼75% symptom severity (16–20 dai); Stage 5∶100% symptom severity (21–25 dai).

The infected leaves were harvested based on the sampling method described by Miedaner *et al.* which classified the infection stages by observing and quantifying morphological changes on the leaves [30]. For our experiments, infected leaves were divided into five stages displaying symptoms in 0%, ∼25%, ∼50%, ∼75% and 100% of the leaf surface (Fig. 5). These stages were observed in plants 3–6 dai, 7–10 dai, 11–15 dai, 16–20 dai and 21–25 dai, respectively (Fig. 5). DNA was subsequently extracted from the leaves belonging to each of the 5 different stages and subjected to different analyses.

### Quantification of pathogen DNA in infected plants

Real-time PCR is the most widely used method to quantify the amount of pathogen DNA in a given DNA sample [11], [31]–[33]. For the purpose of our experiments, the accuracy of Foc quantification using real-time PCR was validated in several ways. Firstly, quantification was performed using two sets of primers, each set targeting different regions of the Foc genome. Secondly, two standard curves from each of the 2 primer sets were generated by adding known amounts of Foc genomic DNA (20 ng, 10 ng, 5 ng, 1 ng, 0.5 ng and 0.1 ng), while DNA from healthy plants served as negative control. Finally, three replicates of all infected plant samples were analyzed for each specified amount of DNA. The average amount of pathogen DNA was quantified from each primer set and standard deviations were calculated.

**Figure 6:**
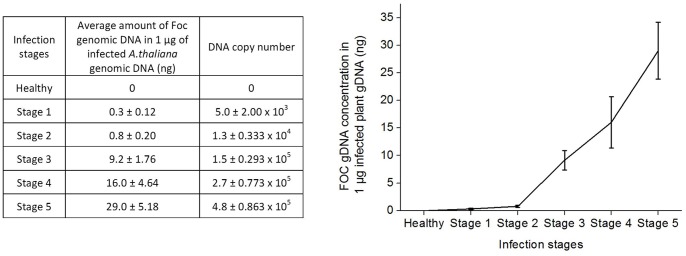
Quantification of Foc genomic DNA in infected *A. thaliana* using real-time PCR. The average amount of Foc genomic DNA present in the infected plant samples were quantified using the 2 primer sets (Foc-F1/Foc-R1 and Foc-F2/Foc-R2) in the real-time PCR.

The standard curves of the two primer sets (Foc-F1/Foc-R1 and Foc-F2/Foc-R2) resulting from linear regression are shown in Fig. S2a and S2b, respectively. Both standard curves indicated a good correlation between the amount of DNA template and the Ct values (R^2^∶0.98 and 0.99, respectively). Melting curve analysis of the PCR amplicons was also performed to confirm successful amplification indicating a maximum Tm of 82.5°C and 85°C, respectively (Fig. S2c and S2d). The amount of Foc genomic DNA in all stages of infection was then determined based on the Ct values and averages of the quantified concentrations from the two primer sets (Fig. 6). Infection in stages 1 and 2 showed very low amounts of Foc genomic DNA while a very significant increase was observed in stage 3 that kept steadily growing until stage 5 (Fig. 6).

### MIP assays on infected plants

**Figure 7:**
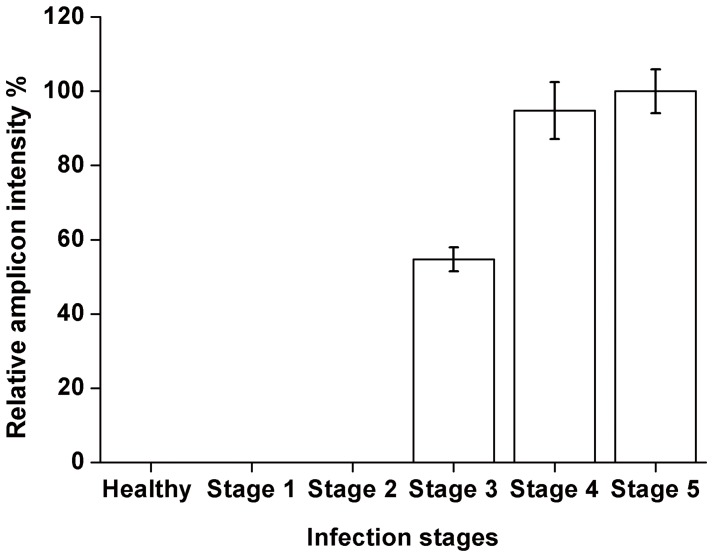
MIP assay on infected *A. thaliana* plant tissue samples. Replicate MIP assays were performed on 2 µg of DNA extracted from the *A. thaliana* leaves belonging to each of the five infection stages as well as non-infected (healthy) leaves. Each reaction contained 2 µg of genomic DNA extracted from infected *A. thaliana* leaves. Non-infected *A. thaliana* was used as negative control. MIP assays were performed in triplicates and amplicon abundance was measured by quantifying the intensity of the DNA bands. Error bars represent standard deviation of the three replicates.

In order to determine if the MIP assay can reproducibly detect the presence of Foc genomic DNA in infected plant tissues, we analyzed DNA from plant samples at different stages of infection as well as non-infected plants. MIPFOC01 was mixed with 2 µg of DNA extracted from plant tissue at each of the five infection stages described earlier (Fig. 5). Following hybridization and gap-fill reaction, the circularized MIPs were amplified by PCR. The PCR amplicons were analyzed using gel electrophoresis and followed by quantifying the intensities of the DNA bands (Fig. 7). No amplification was detected in stages 1 and 2, which according to our RT-PCR quantification (Fig. 6) contained a concentration of pathogen DNA below the limit of detection shown in Fig. 2. However, amplification was observed in stages 3, 4 and 5. The amount of amplified DNA increased gradually from stage 3 to stage 5, although in stage 5 the signal seemed to be close to reaching saturation.

## Discussion

As proof of concept, in this work we optimized the molecular inversion probe assay to detect the plant pathogen Foc in pure cultures and infected *A. thaliana* tissues. The sensitivity, specificity and the dynamic range of the assay has been demonstrated in a singleplex MIP assay. MIPs are highly versatile and can be easily tailored to detect virtually any pathogen in a DNA mixture by carefully designing the MIPs binding domains. After hybridizing to the target DNA, the MIP creates a single stranded gap between the binding domains. This looped physical conformation differentiates it from a variant of the MIP technology which does not generate a single stranded gap between the binding domains known as padlock probes (PLP) [34]–[36]. Padlock probes (PLP) have been developed for several applications including plant pathogen identification [19], [37]–[39]. However, PLPs exhibited limited accuracy when detecting different pathogens from closely related species [39], mostly due to the fact that the specificity of the PLP assay depends on the fidelity of the ligase which has been known to be promiscuous [40]–[42]. Therefore, it is likely that the accuracy of the MIP assay which is defined by a polymerization and a ligation component potentially increased the specificity of pathogen detection.

The binding domains at the 3′ and 5′ end of the MIP are linked by a DNA backbone. This design feature physically restricts the physical distance that can exist in the target genomic DNA for the hybridization of both binding domains, thereby increasing the specificity of the MIPs. In addition, in our approach the signal to noise ratio of the assay was increased by enzymatically digesting the unligated linear MIPs using exonuclease. PCR amplification of the circularized MIPs enabled the MIP assay to detect as little as 2.5 ng of purified Foc genomic DNA. This low detection limit makes the MIP assay an ideal method for detecting pathogens in plants during early stages of infection.

Increasing the relative amount of purified plant DNA to Foc DNA resulted in a decrease in the overall yield of the assay (Fig. 3) suggesting that the MIP hybridized to the target pathogen DNA more efficiently in the presence of lower amounts of plant DNA. The MIP assay was also able to discriminate among three different pathogens, one of which, *Fusarium oxysporum* f.sp. *Lycopersici,* belonging to the same species of Foc (Fig. 4). Based on the sequencing data (fig. 4b), it can be definitively stated that the MIPF0C01 was highly specific in interrogating the target DNA (Foc gDNA). This is further confirmed by figure S1, where in the absence of the Foc gDNA, the MIP failed to generate PCR amplicons in the presence of Fol gDNA and Bc gDNA. Such level of the inherent specificity of MIPs can be attributed to the use of relatively long sequences for target recognition (38 bp) and 48 bp of gap fill-in, but even more important, careful design of the MIP can discriminate between almost identical genomes. In our case we performed *in silico* analysis of the *conglutinans* and *lycopersici* genomes and designed MIPFOC01 to target a region present in Foc but not in Fol. As long as there is enough sequence information available, the same strategy can be used to discriminate between closely related pathogens.

The MIP assay was able to detect the presence of Foc DNA in infected plant tissues from infection stage 3 and forward, while stages 1 and 2 remained undetected. Even though this level of sensitivity is apparently suboptimal, it is mostly due to a very specific characteristic of the pathogen used in our study. In the case of Foc infection of Arabidopsis plants, the appearance of the initial chlorosis symptoms in leaves used for the quantification of the disease development in this work is due to the obstruction of the vascular system in the lower parts of the stem and actually precedes the invasion of the leaves by the fungus [1], [43]. This is a relatively unique characteristic of the infection process used by Foc and does not usually happen in diseases caused by other pathogens, where symptom development is strongly linked to pathogen abundance in the infected tissue. Pathogen quantification by RT-PCR confirmed that the leaves belonging to the infection stages 1 and 2 contained extremely low amounts of Foc that challenged even the detection limit of the RT-PCR (Fig. 6). Roots are nevertheless heavily infected during stages 1 and 2 roots and an obvious question arises as for why do not choose roots as our starting material for the assay. We purposely avoided using roots in our assay to preclude the possibility of interference from Foc present in the soil rather than the plant tissues. Due to the nature of the inoculation method, a large amount of Foc is present in the soil and even after extensive washing, contaminating traces of Foc in the root samples could lead to false positive results. As mentioned above, in most crop diseases the development of symptoms is strongly correlated with the presence of the pathogen and it is therefore expected that the MIP assay method will be able to detect the pathogen and the initial stages of disease development allowing for an early diagnosis.

In conclusion, we have presented here the MIP technology to detect plant pathogens in infected plants. This method can be a reliable alternative to the existing pathogen detection and identification methods such as morphological identifications which involves traditional *in vitro* culturing and isolation methods. The important advantage of the MIP technology over other available methods is its innate capability for multiplexing using molecular inversion probes as have been previously demonstrated in various clinical studies [23], [44]–[47]. The tailor made backbone of the MIPs provide the opportunity to incorporate barcode sequences unique for each probe that can be coupled to other technologies to differentiate thousands of amplifications products in a single step [48]–[50]. Therefore this assay has the potential to be developed into a comprehensive detection system of thousands of pathogens in a single assay, although due to its technical complexity it is unlikely that it will be deployed into a portable device.

## Supporting Information

Figure S1 **Specificity test of the MIP assay.** MIP assay was performed on 50 ng of genomic DNA from three infectious plant pathogens (Foc, Fol and Bc) using the MIPFOC01 probe. The MIP products were analyzed using gel electrophoresis.
(TIF)

Figure S2 **Quantification of Foc genomic DNA in infected** ***A. thaliana*** **using real-time PCR.** a, b) Standard curves using 2 primer sets Foc-F1/Foc-R1 (2a) and Foc-F2/Foc-R2 (2b) and known concentrations of purified Foc genomic DNA (20 ng –100 pg) (blue dots). The concentration of Foc genomic DNA in 5 infection stages as well as in non-inoculated plants was calculated by interpolating on the standard curves (red dots). c, d) Melting curve profiles of the PCR amplicons generated by the 2 primer sets in a real-time PCR.
(TIF)

## References

[1] Agrios GN (2005) Plant pathology. Amsterdam; Boston: Elsevier Academic Press. xxiii, 922 p. p.

[2] Punja ZK, De Boer S, Sanfaçon Hln (2007) Biotechnology and plant disease management. Wallingford Oxfordshire, UK; Cambridge, MA: Cabi Pub. xi, 574 p. p.

[3] Horsfall JG, Cowling EB (1977) Plant disease : an advanced treatise. New York: Academic Press.

[4] Comstock JC (1992) Detection of the sugarcane leaf scald pathogen, Xanthomonas-albilineans, using tissue blot immunoassay, ELISA, and Isolation Techniques. Plant Disease 76: 1033–1035.

[5] Kokoskova B, Janse JD (2009) Enzyme-linked immunosorbent assay for the detection and identification of plant pathogenic bacteria (in particular for *Erwinia amylovora* and *Clavibacter michiganensis* subsp. *sepedonicus*). Methods Mol Biol 508: 75–87. 19301748 10.1007/978-1-59745-062-1_7

[6] Novakova S, Klaudiny J, Kollerova E, Subr ZW (2006) Expression of a part of the Potato virus A non-structural protein P3 in Escherichia coli for the purpose of antibody preparation and P3 immunodetection in plant material. Journal of Virological Methods 137: 229–235. 16876262 10.1016/j.jviromet.2006.06.020

[7] Malin EM, Roth DA, Belden EL (1983) Indirect Immunofluorescent Staining for Detection and Identification of Xanthomonas-campestris Pv Phaseoli in Naturally Infected Bean Seed. Plant Disease 67: 645–647.

[8] Franken AAJM, Zilverentant JF, Boonekamp PM, Schots A (1992) Specificity of polyclonal and monoclonal-antibodies for the identification of Xanthomonas-campestris Pv campestris. Netherlands Journal of Plant Pathology 98: 81–94.

[9] Ward E, Foster SJ, Fraaije BA, McCartney HA (2004) Plant pathogen diagnostics: immunological and nucleic acid-based approaches. Annals of Applied Biology 145: 1–16.

[10] Vincelli P, Tisserat N (2008) Nucleic acid-based pathogen detection in applied plant pathology. Plant Disease 92: 660–669. 10.1094/PDIS-92-5-0660 30769590

[11] Lievens B, Brouwer M, Vanachter ACRC, Cammue BPA, Thomma BPHJ (2006) Real-time PCR for detection and quantification of fungal and oomycete tomato pathogens in plant and soil samples. Plant Science 171: 155–165.

[12] Price JA, Smith J, Simmons A, Fellers J, Rush CM (2010) Multiplex real-time RT-PCR for detection of Wheat streak mosaic virus and Tritcum mosaic virus. Journal of Virological Methods 165: 198–201. 20138086 10.1016/j.jviromet.2010.01.019

[13] Araujo R, Amorim A, Gusmao L (2012) Diversity and specificity of microsatellites within Aspergillus section Fumigati. BMC Microbiology 12: 154. 22838495 10.1186/1471-2180-12-154 PMC3438126

[14] Dai J, Peng H, Chen W, Cheng J, Wu Y (2013) Development of multiplex real-time PCR for simultaneous detection of three Potyviruses in tobacco plants. J Appl Microbiol 114: 502–508. 23164070 10.1111/jam.12071

[15] Markoulatos P, Siafakas N, Moncany M (2002) Multiplex polymerase chain reaction: a practical approach. J Clin Lab Anal 16: 47–51. 11835531 10.1002/jcla.2058 PMC6808141

[16] Sint D, Raso L, Traugott M (2012) Advances in multiplex PCR: balancing primer efficiencies and improving detection success. Methods Ecol Evol 3: 898–905. 23549328 10.1111/j.2041-210X.2012.00215.x PMC3573865

[17] Tooley PW, Bunyard BA, Carras MM, Hatziloukas E (1997) Development of PCR primers from internal transcribed spacer region 2 for detection of Phytophthora species infecting potatoes. Appl Environ Microbiol 63: 1467–1475. 9097445 10.1128/aem.63.4.1467-1475.1997 PMC168442

[18] Lee Young-Mi, Choi YK, Min BR (2000) PCR-RFLP and sequence analysis of the rDNA ITS region in the Fusarium spp. Journal of Microbiology 38: 66–73.

[19] Szemes M, Bonants P, de Weerdt M, Baner J, Landegren U, et al (2005) Diagnostic application of padlock probes-multiplex detection of plant pathogens using universal microarrays. Nucleic Acids Research 33(8): e70. 15860767 10.1093/nar/gni069 PMC1087788

[20] Chillali M, Idder-Ighili H, Guillaumin JJ, Mohammed C, Escarmant BL, et al (1998) Variation in the ITS and IGS regions of ribosomal DNA among the biological species of European Armillaria. Mycological Research 102: 533–540.

[21] Hardenbol P, Baner J, Jain M, Nilsson M, Namsaraev EA, et al (2003) Multiplexed genotyping with sequence-tagged molecular inversion probes. Nature Biotechnology 21: 673–678. 10.1038/nbt821 12730666

[22] Wang YK, Moorhead M, Karlin-Neumann G, Falkowski M, Chen CN, et al (2005) Allele quantification using molecular inversion probes (MIP). Nucleic Acids Research 33 (21): e183. 10.1093/nar/gni177 PMC1301601 16314297

[23] Diep D, Plongthongkum N, Gore A, Fung HL, Shoemaker R, et al (2012) Library-free methylation sequencing with bisulfite padlock probes. Nat Methods 9: 270–272. 22306810 10.1038/nmeth.1871 PMC3461232

[24] Cazzonelli CI, Cavallaro AS, Botella JR (1998) Cloning and characterisation of ripening-induced ethylene biosynthetic genes from non-climacteric pineapple (Ananas comosus) fruits. Australian Journal of Plant Physiology 25: 513–518.

[25] Doyle JJ, Doyle JL (1990) Isolation of plant DNA from fresh tissue. Focus 12: 13–15.

[26] Yamazaki W, Seto K, Taguchi M, Ishibashi M, Inoue K (2008) Sensitive and rapid detection of cholera toxin-producing Vibrio cholera using a loop-mediated isothermal amplification. BMC Microbiology 8: 94. 18547441 10.1186/1471-2180-8-94 PMC2446398

[27] Ge Z, Qing Y, Zicheng S, Shiying S (2013) Rapid and sensitive diagnosis of *Acanthamoeba keratitis* by loop-mediated isothermal amplification. Clin Microbiol Infect 19: 1042–1048. 23413965 10.1111/1469-0691.12149

[28] Clark JS, Hersh MH (2009) Inference in incidence, infection, and impact: Co-infection of multiple hosts by multiple pathogens. Bayesian Analysis 4: 337–365.

[29] Trusov Y, Chakravorty D, Botella JR (2013) *Fusarium oxysporum* infection assays in Arabidopsis. Methods Mol Biol 1043: 67–72. 23913036 10.1007/978-1-62703-532-3_7

[30] Miedaner T, Gang GR, Geiger HH (1996) Quantitative-genetic basis of aggressiveness of 42 isolates of *Fusarium culmorum* for winter rye head blight. Plant Disease 80: 500–504.

[31] Heid CA, Stevens J, Livak KJ, Williams PM (1996) Real time quantitative PCR. Genome Research 6: 986–994. 8908518 10.1101/gr.6.10.986

[32] Klosterman SJ (2012) Real-Time PCR for the quantification of fungi in planta. Plant Fungal Pathogens: Methods and Protocols 835: 121–132. 10.1007/978-1-61779-501-5_8 22183651

[33] Lin YH, Su CC, Chao CP, Chen CY, Chang CJ, et al (2013) A molecular diagnosis method using real-time PCR for quantification and detection of *Fusarium oxysporum* f. sp *cubense* race 4. European Journal of Plant Pathology 135: 395–405.

[34] Nilsson M, Malmgren H, Samiotaki M, Kwiatkowski M, Chowdhary BP, et al (1994) Padlock probes: circularizing oligonucleotides for localized DNA detection. Science 265: 2085–2088. 7522346 10.1126/science.7522346

[35] Hirani R, Connolly AR, Putral L, Dobrovic A, Trau M (2011) Sensitive quantification of somatic mutations using molecular inversion probes. Analytical Chemistry 83: 8215–8221. 21942816 10.1021/ac2019409

[36] Palanisamy R, Connolly AR, Trau M (2011) Epiallele quantification using molecular inversion probes. Anal Chem 83: 2631–2637. 21401108 10.1021/ac103016n

[37] van Doorn R, Szemes M, Bonants P, Kowalchuk GA, Salles JF, et al (2007) Quantitative multiplex detection of plant pathogens using a novel ligation probe-based system coupled with universal, high-throughput real-time PCR on OpenArrays (TM). BMC Genomics 8(1): 276. 17697351 10.1186/1471-2164-8-276 PMC2064939

[38] van Doorn R, Slawiak M, Szemes M, Dullemans AM, Bonants P, et al (2009) Robust detection and identification of multiple oomycetes and fungi in environmental samples by using a novel cleavable padlock probe-based ligation detection assay. Applied and Environmental Microbiology 75: 4185–4193. 19395562 10.1128/AEM.00071-09 PMC2698351

[39] Sikora K, Verstappen E, Mendes O, Schoen C, Ristaino J, et al (2012) A Universal microarray detection method for identification of multiple Phytophthora spp. using padlock probes. Phytopathology 102: 635–645. 22568817 10.1094/PHYTO-11-11-0309

[40] Wu DY, Wallace RB (1989) Specificity of the nick-closing activity of bacteriophage-T4 DNA-ligase. Gene 76: 245–254. 2753355 10.1016/0378-1119(89)90165-0

[41] Xu YZ, Kool ET (1999) High sequence fidelity in a non-enzymatic DNA autoligation reaction. Nucleic Acids Research 27: 875–881. 9889286 10.1093/nar/27.3.875 PMC148260

[42] Cherepanov A, Yildirim E, de Vries S (2001) Joining of short DNA oligonucleotides with base pair mismatches by T4 DNA ligase. Journal of Biochemistry 129: 61–68. 11134958 10.1093/oxfordjournals.jbchem.a002837

[43] Mauchmani B, Slusarenko AJ (1994) Systemic acquired-resistance in Arabidopsis thaliana Induced by a predisposing infection with a pathogenic isolate of Fusarium oxysporum. Molecular Plant-Microbe Interactions 7: 378–383.

[44] Li JB, Gao Y, Aach J, Zhang K, Kryukov GV, et al (2009) Multiplex padlock targeted sequencing reveals human hypermutable CpG variations. Genome Res 19: 1606–1615. 19525355 10.1101/gr.092213.109 PMC2752131

[45] Turner EH, Ng SB, Nickerson DA, Shendure J (2009) Methods for genomic partitioning. Annu Rev Genomics Hum Genet 10: 263–284. 19630561 10.1146/annurev-genom-082908-150112

[46] Lin S, Wang W, Palm C, Davis RW, Juneau K (2010) A molecular inversion probe assay for detecting alternative splicing. BMC Genomics 11: 712. 21167051 10.1186/1471-2164-11-712 PMC3022918

[47] Deng J, Shoemaker R, Xie B, Gore A, LeProust EM, et al (2009) Targeted bisulfite sequencing reveals changes in DNA methylation associaed with nuclear reprogramming. Nat. Biotechnol 27 (4): 353–360. 10.1038/nbt.1530 PMC2715272 19330000

[48] Akhras MS, Thiyagarajan S, Villablanca AC, Davis RW, Nyren P, et al (2007) PathogenMip assay: a multiplex pathogen detection assay. PLoS One 2: e223. 17311101 10.1371/journal.pone.0000223 PMC1794193

[49] Hyman RW, Richard W, Davis RW, St Onge RP, Kim H, et al (2012) Molecular probe technology detects bacteria without culture. BMC Microbiology 12 (1): 29. 10.1186/1471-2180-12-29 PMC3316761 22404909

[50] Novais RC, Borsuk S, Dellagostin OA, Thorstenson YR (2008) Molecular inversion probes for sensitive detection of Mycobacterium tuberculosis. Journal of Microbiological Methods 72: 60–66. 18036684 10.1016/j.mimet.2007.10.009

