# Supplementary figures and images for: Molecular Inversion Probe: A New Tool for Highly Specific Detection of Plant Pathogens
Source: PLoS One. 2014 Oct 24;9(10):e111182. doi: 10.1371/journal.pone.0111182 (PMC4208852; doi:10.1371/journal.pone.0111182)

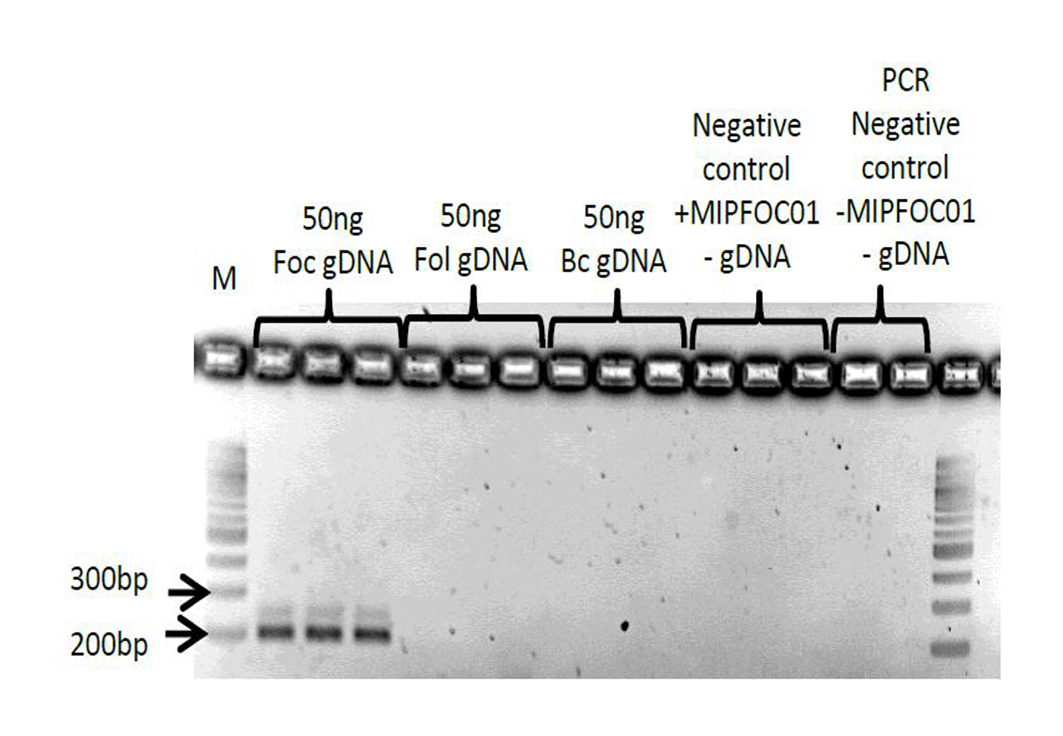

Supplement: Figure S1 — Specificity test of the MIP assay. MIP assay was performed on 50 ng of genomic DNA from three infectious plant pathogens (Foc, Fol and Bc) using the MIPFOC01 probe. The MIP products were analyzed using gel electrophoresis. (TIF) [file pone.0111182.s001.tif]

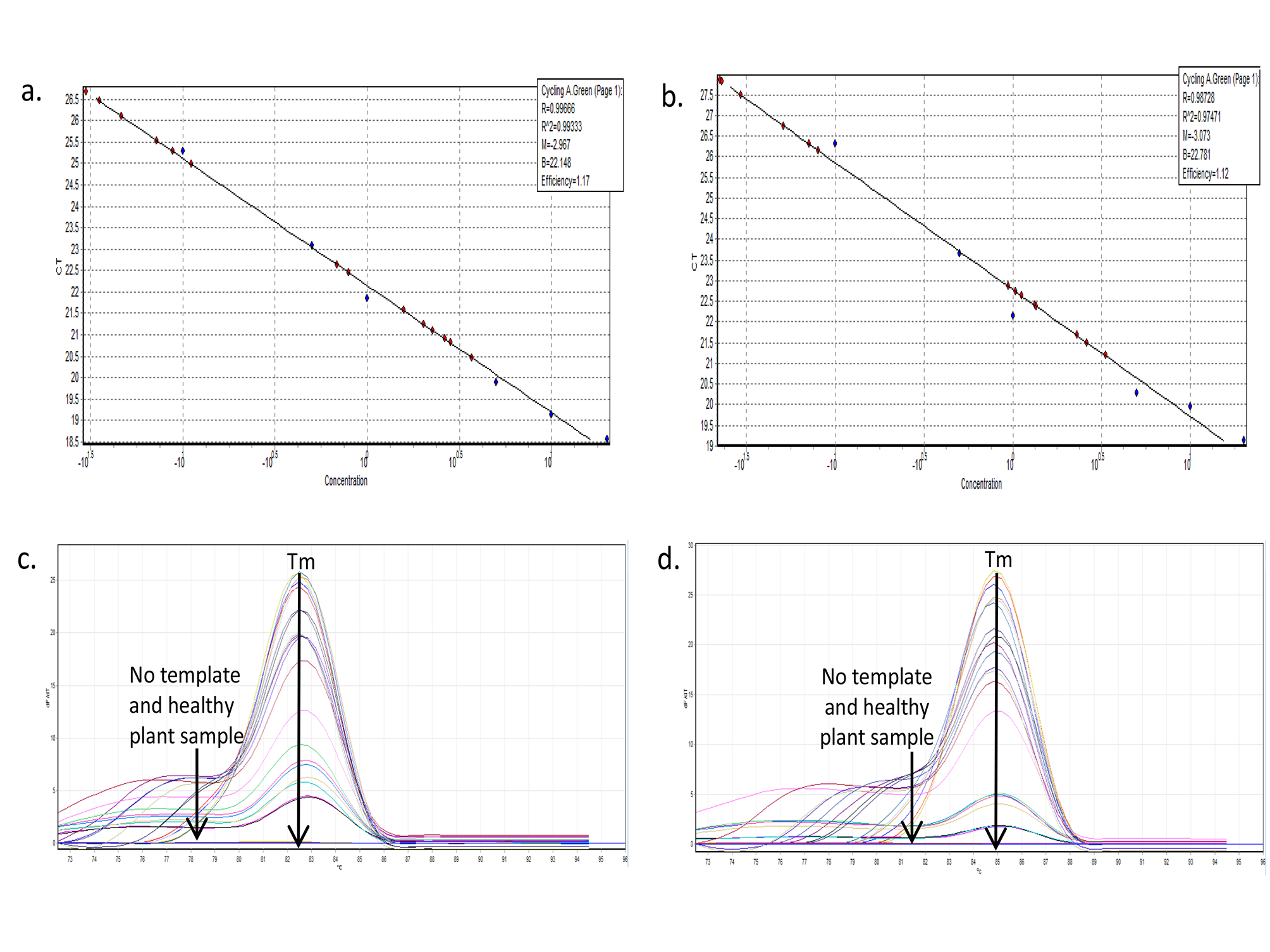

Supplement: Figure S2 — Quantification of Foc genomic DNA in infected A. thaliana using real-time PCR. a, b) Standard curves using 2 primer sets Foc-F1/Foc-R1 (2a) and Foc-F2/Foc-R2 (2b) and known concentrations of purified Foc genomic DNA (20 ng –100 pg) (blue dots). The concentration of Foc genomic DNA in 5 infection stages as well as in non-inoculated plants was calculated by interpolating on the standard curves (red dots). c, d) Melting curve profiles of the PCR amplicons generated by the 2 primer sets in a real-time PCR. (TIF) [file pone.0111182.s002.tif]
